# Supplementary material for: Association between oral microbiome and seven types of cancers in East Asian population: a two-sample Mendelian randomization analysis
Source: Front Mol Biosci. 2023 Nov 21;10:1327893. doi: 10.3389/fmolb.2023.1327893 (PMC10702768; doi:10.3389/fmolb.2023.1327893)
Supplement: Supplementary file 1 [file DataSheet1.ZIP › Supplementary Materials/MR plots of saliva/saliva═╝/Pancreatic cancer/pheno.2346_to_pancreatic cancer_forest.pdf]

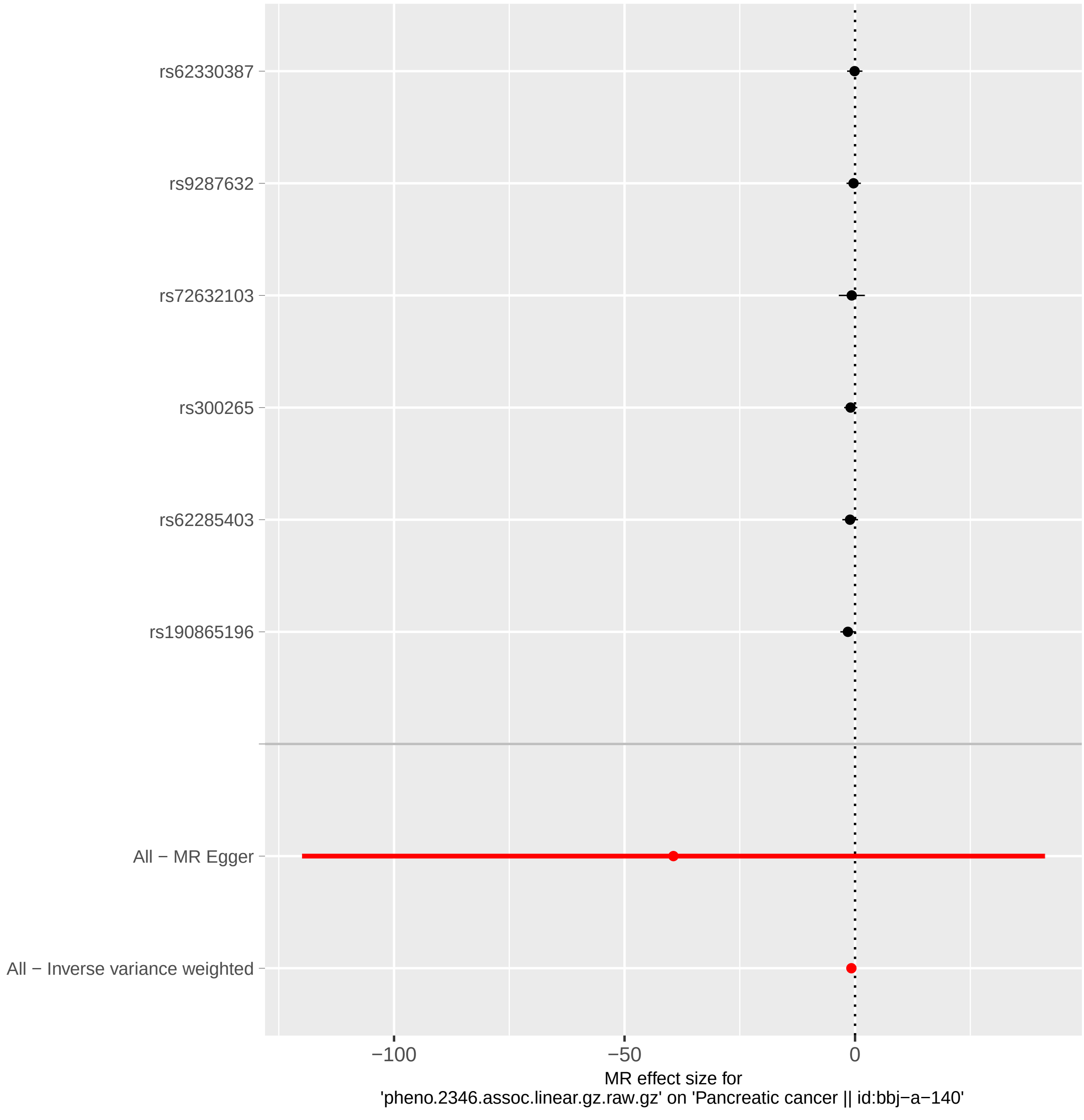

rs62330387
rs9287632
rs72632103
rs300265
rs62285403
rs190865196
All − MR Egger
All − Inverse variance weighted
−100
−50
0
MR effect size for
'pheno.2346.assoc.linear.gz.raw.gz' on 'Pancreatic cancer || id:bbj−a−140'
